# Supplementary material for: Causal Relationship between Aging and Anorexia Nervosa: A White-Matter-Microstructure-Mediated Mendelian Randomization Analysis
Source: Biomedicines. 2024 Aug 16;12(8):1874. doi: 10.3390/biomedicines12081874 (PMC11351342; doi:10.3390/biomedicines12081874)
Supplement: Supplementary file 1 [file biomedicines-12-01874-s001.zip › Supplementary Figure/Figure S4/Singe SNP plot of MR analysis for TL to ubm-b-1684.pdf]

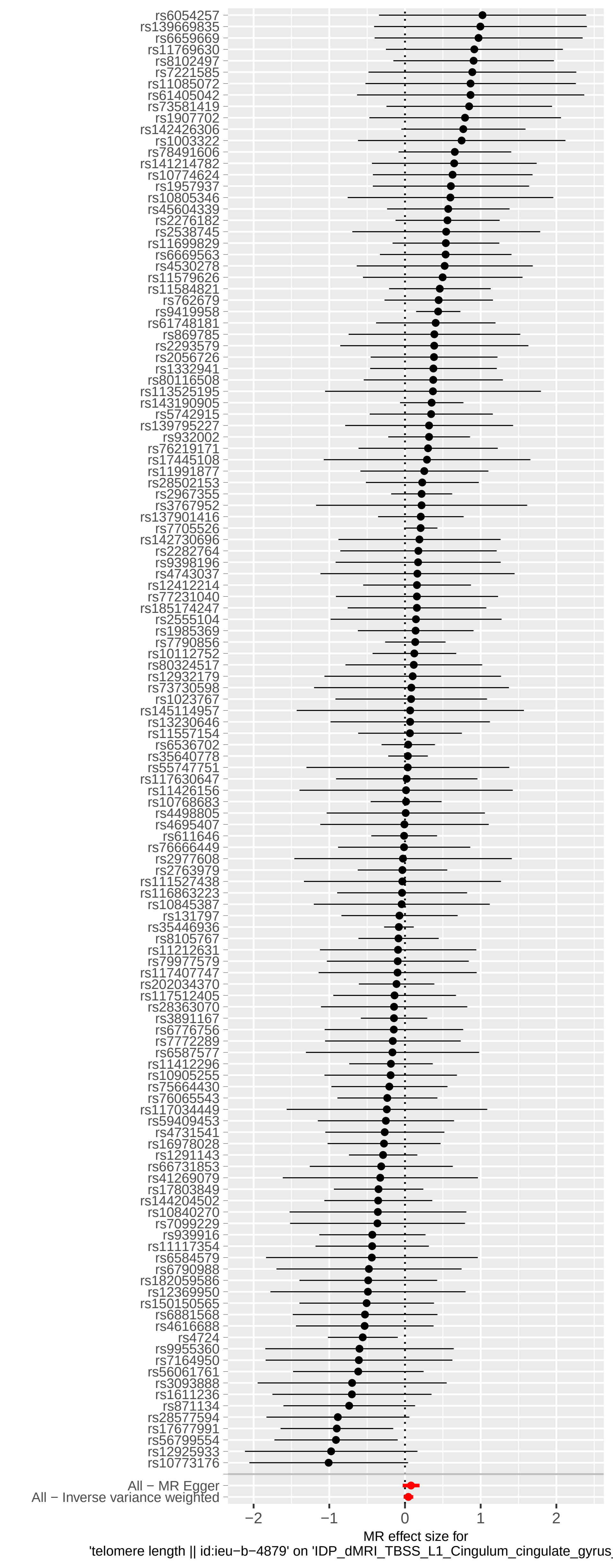
rs6054257
rs139669835
rs6659669
rs11769630
rs8102497
rs7221585
rs11085072
rs61405042
rs73581419
rs1907702
rs142426306
rs1003322
rs78491606
rs141214782
rs10774624
rs1957937
rs10805346
rs45604339
rs2276182
rs2538745
rs11699829
rs6669563
rs4530278
rs11579626
rs11584821
rs762679
rs9419958
rs61748181
rs869785
rs2293579
rs2056726
rs1332941
rs80116508
rs113525195
rs143190905
rs5742915
rs139795227
rs932002
rs76219171
rs17445108
rs11991877
rs28502153
rs2967355
rs3767952
rs137901416
rs7705526
rs142730696
rs2282764
rs9398196
rs4743037
rs12412214
rs77231040
rs185174247
rs2555104
rs1985369
rs7790856
rs10112752
rs80324517
rs12932179
rs73730598
rs1023767
rs145114957
rs13230646
rs11557154
rs6536702
rs35640778
rs55747751
rs117630647
rs11426156
rs10768683
rs4498805
rs4695407
rs611646
rs76666449
rs2977608
rs2763979
rs111527438
rs116863223
rs10845387
rs131797
rs35446936
rs8105767
rs11212631
rs79977579
rs117407747
rs202034370
rs117512405
rs28363070
rs3891167
rs6776756
rs7772289
rs6587577
rs11412296
rs10905255
rs75664430
rs76065543
rs117034449
rs59409453
rs4731541
rs16978028
rs1291143
rs66731853
rs41269079
rs17803849
rs144204502
rs10840270
rs7099229
rs939916
rs11117354
rs6584579
rs6790988
rs182059586
rs12369950
rs150150565
rs6881568
rs4616688
rs4724
rs9955360
rs7164950
rs56061761
rs3093888
rs1611236
rs871134
rs28577594
rs17677991
rs56799554
rs12925933
rs10773176
All − MR Egger
All − Inverse variance weighted
−2
−1
0
1
2
MR effect size for
'telomere length || id:ieu−b−4879' on 'IDP_dMRI_TBSS_L1_Cingulum_cingulate_gyrus_
